# Retrospective review of utilisation and outcomes of diaphragmatic EMG monitoring and neurally adjusted ventilatory assist in a central London teaching hospital over a 3-year period

**DOI:** 10.1186/cc12084

**Published:** 2013-03-19

**Authors:** A Skorko, D Hadfield, A Vercueil, C Bell, A Feehan, K Peters, P Hopkins

**Affiliations:** 1 King's Health Partners AHSC, London, UK

## Introduction

The theoretical advantages of monitoring the electrical activity of the diaphragm (EAdi) and neural triggering of support breaths (NAVA-Maquet) have not yet been shown to translate into significant clinical benefit [1]. Here we assess the effect of EAdi monitoring, in patients at risk of prolonged weaning, on outcomes.

## Methods

Institutional ethics approval was obtained. The Medtrack clinical information system was searched to identify patients who received MV for >48 hours and who had significant chronic pulmonary disease or left/right ventricular impairment between April 2009 and March 2012. Age, APACHE II score, ventilated days, time in NAVA and outcome were compared between groups who had or had not received EAdi monitoring.

## Results

**Figure 1:**
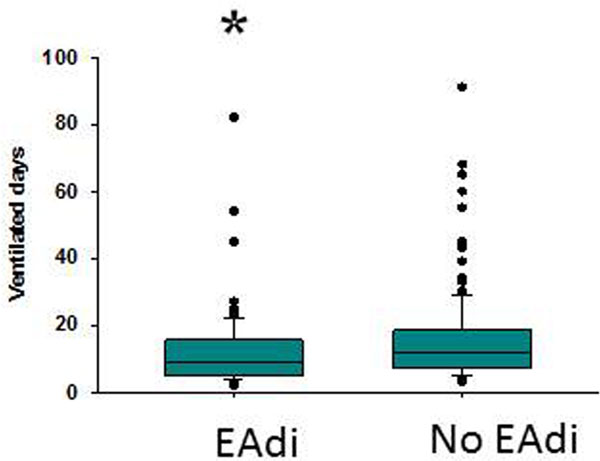


In total, 493/2,684 (18.3%) patients had heart-lung risk factors for prolonged weaning. One hundred and four patients received EAdi monitoring. Ventilated days were significantly reduced (Figure 1) in the EAdi monitored group (median 9) versus the nonmonitored group (median 12); *P *= 0.024 (Mann-Whitney U test). ICU mortality was not significantly different and there was no correlation between time spent in NAVA and days ventilated.

## Conclusion

EAdi catheter insertion was associated with a significant reduction in time spent on MV. A prospective RCT will be needed to confirm benefit and explore mechanisms.
